# Efficacy of *In Ovo* Delivered Prebiotics on Growth Performance, Meat Quality and Gut Health of Kuroiler Chickens in the Face of a Natural Coccidiosis Challenge

**DOI:** 10.3390/ani9110876

**Published:** 2019-10-28

**Authors:** Harriet Angwech, Siria Tavaniello, Acaye Ongwech, Archileo N. Kaaya, Giuseppe Maiorano

**Affiliations:** 1 Department of Agricultural, Environmental and Food Sciences, University of Molise, 86100 Campobasso, Italy; siria.tavaniello@unimol.it (S.T.); oacaye@yahoo.com (A.O.); maior@unimol.it (G.M.); 2 Department of Biology, Faculty of Science, Gulu University, P.O. Box 166 Gulu, Uganda; 3 School of Food Technology, Nutrition and Bio-engineering, Makerere University, P.O. Box 7062 Kampala, Uganda; kaaya.archileo48@gmail.com

**Keywords:** Kuroiler chickens, in ovo prebiotics, performance traits, fatty acids, coccidiosis

## Abstract

**Simple Summary:**

The management of coccidiosis in poultry farms is mainly dependent on the use of anticoccidial drugs. Development of resistance to existing anticoccidial drugs coupled with restrictive use of antibiotics to control secondary bacterial infections following the ban on antibiotics, stresses the urgent need to explore alternative strategies for maintaining intestinal functionality in chickens for improved productivity. Prebiotics have been proposed as a solution to the intestinal problems of poultry. This study demonstrates that in ovo delivered prebiotics with or without antibiotics reduces severity of intestinal lesions and oocyst excretion induced by natural infection with *Eimeria*. Prebiotics protected Kuroiler chickens from coccidia in particular in the first 56 days of age and tended to have a synergistic effect with anticoccidial drug in the management of the disease post-infection in the field, with positive effects on performance and meat quality.

**Abstract:**

A study was carried out to assess the efficacy of prebiotic delivered in ovo on performance, carcass traits, meat quality and gut health in the face of a natural coccidiosis infection in Kuroiler chickens. On d 12 of incubation, 150 fertile eggs were divided into a prebiotic group injected with trans-galactooligosaccharides (Bi^2^tos) and a control group uninjected. Hatched chicks from each group were further divided: One group received antibiotic chick formula while the other was left untreated, giving rise to 4 groups—Control (C), Antibiotic (A), Bi^2^tos (B), and Bi^2^tos + Antibiotic (AB). Prebiotic improved growth performance at six weeks of age, AB birds were the heaviest at the end of the rearing period. The highest intestinal lesion scores and oocyst counts were recorded in C birds. B group had a slightly higher carcass weight and cuts yields tended to be higher in treated groups compared to C. Meat from B group displayed a higher amount of polyunsaturated fatty acids compared to C and a positively lower n-6/n-3 ratio compared to C and A. In conclusion, prebiotics with or without antibiotics reduced severity of intestinal lesions and oocyst excretion induced by natural infection with *Eimeria*, with positive effects on Kuroiler chicken productive traits.

## 1. Introduction

Poultry production forms an integral part of the economy with many socio-economic and cultural values attached to the birds in most developing countries including Uganda. However, one of the major challenges to improved poultry production is the prevalence of poultry diseases that threaten the intensification of production. Among these are gastrointestinal parasitic diseases notably, coccidiosis. Coccidiosis is a disease caused by parasites of the genus *Eimeria* and phylum Apicomplexa with a complex life cycle, affecting mainly the intestinal tract of many species of birds [1]. It is of great economic significance in farm animals, especially chickens. The economic significance of coccidiosis is attributed to decreased animal production in terms of poor feed conversion, growth depression and increased mortality; and the costs involved in treatment and prevention. Pathogenesis entails *Eimeria* invading the intestinal cells as part of the life cycle. The resulting intestinal damage impairs nutrients digestion and absorption, gut barrier function, and ultimately leads to bacterial infections particularly necrotic enteritis [2]. The global annual costs inflicted by coccidiosis to commercial poultry have been estimated at 2 billion € [1].

For the past 5 decades, the use of anti-coccidial feed additives has played a major role in the growth of the poultry industry facilitating increased availability of quality and affordable poultry products to the consumers. However, some degree of resistance to all anticoccidial drugs, including ionophores which are now the mainstay of coccidiosis control has been reported [3,4]. Concerns over the development of resistant *Eimeria* species to existing anticoccidial drugs and restrictive use of antibiotics to control secondary bacterial infections further stresses the urgent need to explore alternative strategies for maintaining intestinal functionality in chickens. In addition, serious public health and food safety concerns regarding drug residues in animal products highlight the need for researchers to develop alternative strategies for the control of parasitic problems [5,6]. Vaccines for coccidiosis have been reported as an effective tool for disease control [7] and to ameliorate anticoccidial drug resistance in poultry [4]. However, their efficacy depends greatly on the management strategies in a farm.

The digestive tract of animals harbors a great number of living and metabolizing microorganisms (microbiota), that not only influence physiological functions of the host, but are also considered fundamental for a proper development of several vital traits, including immune system [8]. Thus, the past decades, have seen much effort going into optimizing the gut microbiota of chickens using dietary interventions [9]. Although use of antibiotics at subtherapeutic levels has been the most popular and perhaps the most effective strategy to enhance feed efficiency and to keep animals healthy, the approach is no longer a feasible tool for poultry production performance enhancement due to its ban by the EU in 2006 [9]. Incorporation of immunobiotics, particularly lactic acid bacteria is thought to be useful as immunomodulators to stimulate the gut-associated immune system in neonatal chicks, and thereby protect them from disease without decreasing growth performance as a possible substitution of antibiotics [10]. Prebiotics, which are defined as non-digestible oligosaccharides, are also potent modulators of the intestinal microflora [11]. For instance, addition of prebiotic mannanoligosaccharides (MOS) to the diet of broilers reduced the severity of the infection due to either *E. tenella* alone [12] or a mixture of *E. acervulina*, *E. maxima*, and *E. tenella* [13]. However, for the bioactives to be effective, these compounds have to be administered to the animals under fully controlled conditions and as early as possible. An innovative method for introducing bioactive substances into chickens is the in ovo injection into eggs intended for hatching. This is a technique that is based on the introduction on the appropriate day of embryonic development of bioactive substances into the air chamber of the egg or directly into the developing embryo [14]. This method allows for a precise and uniform delivery of the bioactive substance to all embryos at an early stage of development, which unifies the effects of prebiotics across the flock and ensures proper development of gut microflora in all chicks [15].

Studies conducted in the temperate climatic condition have already revealed that in ovo injection of prebiotics and probiotics into the air cell during embryogenesis improves egg hatchability [15] and modulate the optimal composition of the chicken’s microbiota, fully developed at hatching [14,16,17,18]. These effects are reportedly stable throughout the chicken’s lifespan, influencing metabolic and immune responses of the host, resulting in improvement in performance and meat quality [19,20,21].

However, the efficacy of in ovo delivered prebiotics has been evaluated mainly under fully controlled disease-free experimental conditions in the temperate using meat type chickens [14,19,20,21,22]. In this study, it was hypothesized that prebiotics perform differently under field conditions in the tropics where management and environmental factors predispose birds to enteric diseases. Thus, the efficacy of in ovo delivered prebiotics, antibiotic-chick formula, and a combination of the two on growth performance, carcass traits, meat quality, and gut health in the face of a natural coccidiosis infection was assessed in Kuroiler chickens reared under field conditions in Uganda.

## 2. Materials and Methods

### 2.1. Birds and Experimental Design

To evaluate the efficacy of prebiotics delivered in ovo on productive and meat quality traits and gut health under tropical climatic conditions, 200 eggs from Kuroiler chickens were incubated in a commercial broiler hatchery. On the 12th day of incubation eggs were candled and 150 eggs with viable embryos were randomly divided into two groups. Of these, 75 eggs were injected (into the air chamber) with 200 μL of physiological saline solution containing a commercial prebiotic (trade name: Bi^2^tos, Clasado Biosciences Ltd., Jersey, UK) at dose of 3.5 mg/embryo, a non-digestive trans-galacto oligosaccharides (GOS) from lactose digested with *Bifidobacterium bifidum* NCIMB 41171. Immediately after injection, all holes in the eggs were sealed with natural glue. The eggs were injected on d 12 of embryonic incubation when the allantochorion is completely developed and highly vascularized [23]. The rest of the eggs were left uninjected as control. The egg incubation was continued until hatching.

At hatching, a total of 96 healthy one day old chicks (40.0 g average weight) (24 males (M) and 24 females (F) from control and Bi^2^tos groups) were vaccinated against Marek’s disease, and transferred to the experimental farm as required where they were reared in a brooder house for 21 days before being transferred to littered floor pens with rice husk. Hatched chicks from each of the 2 experimental groups above were further randomly divided into 2 subgroups: One group received antibiotic chick formula (poltricin with oxytetracycline at a dose of 1 g/L of drinking water for 7 days) while the other was left untreated. Thus, giving rise to 4 experimental groups: Control (C, n = 24), Antibiotic (A, n = 24), Bitos (B, n = 24), and Bitos + Antibiotics (AB, n = 24). The birds (n = 3 replicate/group, 8 birds in each replication, 4 M and 4 F) were reared in a local poultry farm in the Gulu District where coccidiosis infection was previously confirmed by field veterinarians (personal communication with the District Veterinary Officer) so that the birds pick the infections naturally from contaminated litter. Routine hygiene practices such as fumigation and litter changing were observed during the rearing period as recommended. All birds were reared under semi-intensive confined system for a period of 18 weeks during the wet season (mid-October to November) and dry season (December to February) at stocking density of 0.3 m^2^/bird for indoor area and 15 m^2^/bird for outdoor area. Birds had outdoor access from the pens provided during daylight hours (from 7.00 am to 5.00 pm) and were exposed to natural environment (with average temperature ranging from 19–32 °C and pasture). Birds were confined to indoor pens at night. The management procedure was the same in both sections of the house. Strict biosecurity procedures were maintained between treatment groups to minimize cross-contamination between pens of different treatment groups. Chickens were fed ad libitum starter, grower, and finisher diets (Table 1) supplemented with pasture. The birds had constant access to water. Body weights were taken per pen on a weekly basis. Faecal samples were collected for parasitological analysis to check for possible infection with *Eimeria* parasites along the rearing period. The animals were reared according to the recommendations of poultry policies, legislation and strategies in Uganda [24]. The reference of the ethical commission of Gulu University is UG-REC-017.

### 2.2. Slaughter Survey

At the age of 18 weeks, a total of 24 birds were randomly selected (12/Sex and 6 from each experimental group, 2 from each replicate: 1 male; 1 female), weighed and slaughtered. After evisceration, the hot carcass weight was recorded, and carcass yield was calculated. In addition, the breast muscle, legs, wings, and back + neck were removed from all carcasses and their percentages based on hot carcass weight were calculated. After 24 h of refrigeration, the pectoral muscle (PM) pH was measured, using a portable pH meter equipped with a penetrating glass electrode. Water holding capacity (WHC) was measured on the right PM 24 h after chilling using filter paper (Whatman No.1) press method [25] and was expressed as free water in meat. The left PM was removed, vacuum packaged, and stored frozen (−20 °C) for fatty acids analysis.

### 2.3. Parasitological Analyses-Oocyst Count

The numbers of oocysts in faeces were determined in samples collected from each pen during the rearing period from week 4. For each pen, fresh excreta samples were collected from every corner of the pen and from the center of the pen and were kept in separate airtight plastic bags. The modified McMaster counting chamber technique of Hodgson [26] was used. Briefly, 10% (w/v) faeces suspension in a salt solution (151 g NaCl mixed into 1 L of water) was prepared. After shaking thoroughly to obtain a homogenous mixture, 1 mL of the suspension was mixed with 9 mL of a salt solution (131 g of NaCl mixed into 1 L of water). Then, the suspension was pipetted into a McMaster chamber and the number of oocysts was counted and expressed per gram of faeces as described by Peek and Landman [27].

### 2.4. Lesion Scoring

On weeks 12 (on the day of starting therapeutic treatment with Amprolium) and 18 (at the end of the experimental period) 2 birds/replicate; 6 birds/treatment, were randomly selected and coccidial intestinal lesion scored. The 0–4 lesion scoring system of Johnson and Reid [28] was used. The areas scored were the upper, middle, and the caecal regions of the intestine, which are the natural predilection sites for *Eimeria* spp. of veterinary significance in poultry, considering that in nature mix infection with two or three *Eimeria* spp. is not uncommon. Based upon severity of the lesions, a score of 0 (no lesions), 1 (mild lesions), 2 (moderate lesions), 3 (severe lesions), or 4 (extremely severe lesions) was recorded for each chicken. The severity of coccidial lesions was scored while the investigator was blinded to treatment modality.

### 2.5. Total lipid and Fatty Acid Composition of Muscle

Lipid extraction from PM samples was performed by the method of Folch et al. [29]. The extracted lipids were esterified and analyzed by gas chromatography (GC/FID). Briefly, 1 µL of the esterified extract was injected onto a 30 m × 0.32 mm × 0.5 µm solgel wax column with polyethylene-glycol (PEG) as the stationary phase and helium gas at 20 psi as the mobile phase. The column was mounted in a GC/FID (Varian chrompack CP-3800). The injector temperature was 260 °C. The temperature of the column was kept at 50 °C for 5 min after injection and thereafter increased to 180 °C at a rate of 20 °C/min, followed by an increase of 2 °C/min to 200 °C, held for 11 min and then finally ramped to 250 °C at 2 °C/min, held for 2.5 min. The individual fatty acid peaks were identified by comparison of retention times with those of known standard FAME mixture (Supelco 47885-U; Sigma-Aldrich, St. Louis, MO, USA) containing 37 fatty acids run under the same operating condition. Quantification of the esters was achieved by integration of the peaks using interactive graphics software, with the relative amount of each fatty acid ester in each sample being expressed as a percentage of all the esters in the sample. To assess the nutritional implications, the n-6 fatty acids/n-3 fatty acids and the PUFA/SFA ratios were also calculated.

### 2.6. Statistical Analysis

Statistical analyses of the data were performed using SPSS [30]. Data on in vivo performance were analyzed by one-way ANOVA where treatment was the main factor. Data on slaughter traits and meat quality characteristics were evaluated by factorial ANOVA where treatment and sex were the main factors. Scheffe’s test was applied to compare the mean values among the experimental groups. For in vivo performance, the pen was considered as the experimental unit. For slaughter traits, lesion scores and meat quality, the individual bird was considered as the experimental unit.

## 3. Results and Discussion

### 3.1. In vivo Performance and Gut Health

Hatchability was similar between the two experimental groups. Results of the effect of the treatment on body weight gain (BWG) are presented in Table 2. The mechanisms of action of in ovo injected bioactive substances are complex [17], but researchers still predict their positive effects on growth performance. Generally, the results of the experiment regarding BWG indicated a fluctuating effect among the weeks considered.

Within the first 3 weeks of life, C chickens had higher BWG (+20 g; *p* < 0.05) compared to B group; while intermediate values (*p* > 0.05) were found for A and AB groups. Conversely, from 3 to 6 weeks, the prebiotic improved BWG compared to the rest of the treatment groups (*p* < 0.05 and *p* < 0.01). But for the period 9 to 12 weeks the prebiotic group (B) showed significantly lower BWG than the other groups (*p* < 0.05 and *p* < 0.01). BWG did not differ (*p* > 0.05) among experimental groups for the period 6 to 9 weeks and 12 to 15 weeks, as well as 15 to 18 weeks. The BWG registered for the whole period (1–18 weeks) was the highest (*p* < 0.05) in birds that received prebiotics in ovo with oxytetracycline in water (AB) and lowest (*p* < 0.05) in oxytetracycline group (A), and intermediate values were found for C and B groups (*p < 0.05*). This further confirms the result of Hooge et al. [31], who found the highest final BW in the group that received a combination of feed additives (antibiotics and mannan oligosaccharides). Moreover, Pruszynska-Oszmalek et al. [32] reported a significant increase in final BW (d 34) of birds in ovo injected with inulin or Bi^2^tos. These findings support the hypothesis that prebiotics promote growth in chickens owing to their ability to strongly bind the pathogenic bacteria and decoy pathogens away from the intestinal lining [16]. Differently, Bednarczyk et al. [22] reported improvements in performance for prebiotic-treated birds only in the first 3 weeks of life. Considering that in the present study, slow growing birds were used unlike in previous study [22], probably the effect of genotype and differing managerial and environmental factors as well as the prebiotic type used, could explain the variation in the obtained results. For instance, Hanning et al. [33] assessed growth performance in two breeds of chickens (Naked Neck, a slow growing breed and Cornish White Rock cross broilers) in response to three types of prebiotics (plum fibres, galactooligosaccharides—GOS, and fructooligosaccharides—FOS). Positive effects of GOS were observed only from week 4 to week 6 and no significant difference was recorded earlier than the 4th week or on the 8th week (end of experiment) for Naked Neck birds. While for the Cornish White Rock cross broilers no significant differences were observed but instead the same treatment lowered BW at week 6 compared to other treatments and the control. On the other hand, the authors found no difference between the group that received FOS and the control throughout the experimental period while plum showed significantly higher BW compared to the control only on week 8 in both chicken breeds [33].

As shown in Table 3, the control birds generally excreted more oocysts compared to the other groups (*p* < 0.05 and *p* < 0.01) throughout the observation period. Oocysts have been found starting from the 6th week, with persistence and a progressive increase in the presence of parasites. The highest amount of oocysts shed were observed on week 11 in all experimental groups but were higher in the C and A groups in comparison with the B and AB groups (*p* < 0.05). However, the B and AB groups started showing clinical signs of the disease by week 11, barely two weeks from the first appearance of oocysts in their excreta. The many defense mechanisms involved in the protection from coccidian infection could probably explain the above observation. Most likely the control birds had gained some sort of immunity in the course of the early exposure to the infection compared to the treatment group. Antibodies (IgA, IgG and IgM) production begins shortly after natural infection [34] or vaccination [35] with an efficacious protection of intestinal mucosa and a significant reduction in clinical signs’ severity and mortality rates. It is however important to note that this immunity does not protect the bird for its entire life time. Furthermore, cellular immune response supported by the T lymphocytes enhances resistance to coccidiosis [36] and cytokines intensify the level of this protection. The increase in interferon gamma levels (IFN-γ) is particularly associated with protective immunity against coccidiosis [37]. Since prebiotics have been reported to have immuno-modulatory effects on birds [18], this probably explains the delay in the birds of the prebiotic-treated groups from acquiring infection as early as the controls under the same rearing condition of the experiment.

In the present study, on period 9–12 weeks BWG of the prebiotic group was lower than those of the other experimental groups. This coincided with the onset of appearance of clinical signs of the disease in the same group. Buzkurt et al. [38] observed a general decrease in feed intake in prebiotic treated birds following infection with *Eimeria* compared to the challenged control birds. This probably explains the drop in BWG observed here. However, the response of the birds in the prebiotic groups (A and AB) to treatment with anticoccidial drug was generally better than the control and antibiotics groups as shown by the immediate improvement in BWG and a reduction on oocyst count following the commencement of treatment (weeks 12 to 14; Table 3).

The effect of the treatment on intestinal lesion score is shown in Table 4. The C birds showed numerically higher intestinal lesion scores at week 12 (*p* > 0.05) and more markedly at week 18 (*p* < 0.01) compared to the B and AB groups. Barberis et al. [37] observed better health condition and production performances in birds of the group that received prebiotic-anticoccidial combination in which a decrease in coccidia replication and lesion scores were found in association with low oocysts shedding kinetics and a stimulated immune system (increased lymphoid organs weights, indices and a bigger number of lymphocytes) in *Eimeria* experimentally infected birds. The protective effect of prebiotics against coccidiosis is thought to be related to the inhibition of asexual schizonts’ development following the stimulation of local immune mechanisms [12]. However, several hypotheses have been put forward to explain the beneficial effect of prebiotics in avian coccidiosis control. It is believed that these products have the ability to simultaneously stimulate cellular immune response which plays a major role in controlling intestinal parasitism and local production of secretory antibodies during natural exposure to *Eimeria* [39]. The bursa of fabricius, spleen, thymus, and lymphoid tissue associated to the intestine (cecal tonsils) are known to be major actors in the immune response against intestinal pathogens [34]. Nollet et al. [40] reported that prebiotics (MOS) augment vaccination thereby increasing the resistance of birds to coccidia infection. The inclusion of prebiotics has been shown to enhance the cellular and humoral immune responses [17,18,41] in broilers. Furthermore, they protect the intestinal mucosa against inflammatory reactions induced by pathogens or toxins by increasing the villi length and facilitating their regeneration [42]. Prebiotics can compete with sporozoites for binding sites on intestinal epithelial cells and thereby reduce the adhesion and the subsequent proliferation of parasites [43].

### 3.2. Carcass Traits, and pH and Water Holding Capacity of Breast Muscle

The effects of the treatment and sex on slaughter traits, pH, and water holding capacity are presented in Table 5. Slaughter weight was not significantly different among treatments. Conversely, carcass weight was slightly higher in the B group compared to the rest of the treatments (*p* = 0.027), while carcass yield was similar (*p* > 0.05) among the groups. Breast weight was not affected (*p* > 0.05) by treatment. Differently, breast yield was generally higher in all treatment groups compared to the C, however significant differences were found only with AB group (+6.0%; *p* < 0.05). Legs weight was not significantly different among experimental groups but tended to be higher in B and AB groups (*p* = 0.055). Also, legs yield tended to be higher in treated groups compared to C (*p* = 0.079). Wings weight was higher in B and AB groups (*p* < 0.05) compared to the control, A group showed intermediate value (*p* > 0.05). Wings yield was higher in AB group compared to the C group (*p* < 0.05), intermediate values were observed in A and B groups (*p* > 0.05). In general, back + neck weight was slightly higher (*p* = 0.053) in B and AB groups, while its incidence on the carcass was not affected by the treatment (*p* > 0.05). This is contrary to the results reported in Maiorano et al. [19] but consistent in part with Maiorano et al. [16], who found no significant differences in slaughter weight, carcass weight, and carcass yield but reported a slightly higher pectoral muscle percentage in prebiotics compared to the control group. In slow-growing broilers fed three different types of prebiotics, Hanning et al. [33] did not find any effect on breast and wing yields.

Regarding the effect of sex on the studied carcass traits, as expected, males were heavier (36.3%) than females (*p* < 0.01) with higher carcass and cuts weights (*p* < 0.01 and *p* < 0.05); while carcass and cuts yields were not influenced by sex (*p* > 0.05). Maiorano et al. [19] who studied the effects of different prebiotics (Bi^2^tos and DiNovo) in commercial poultry found that males were heavier (+14.6%, *p* < 0.01), had higher carcass and breast weights (*p* < 0.01) but similar breast muscle yield (*p* > 0.05). Significant interaction between treatment x sex (*p* < 0.05) was observed for breast yield: In the control group males showed higher value compared to females, an opposite trend was observed in other groups. A significant interaction was found also for wings weight: only in A group, males showed a lower wing weight compared to females.

As reported in Table 5, pH and WHC values were similar (*p* > 0.05) among experimental groups and between the two sexes. pH is one of the most important qualitative attributes of meat. Overall, the pH values obtained in this study totally fit within the pH range accepted for commercial poultry meat [44], and although it is not different among experimental groups, agrees with the findings of An et al. [45] and Sirri et al. [46] for slow and medium growing strains of birds slaughtered at more or less the same age. The pH values obtained in this study are however lower than those reported by Eleroglu et al. [47] (5.88–6.24) for birds slaughtered at 14 weeks of age.

### 3.3. Intramuscular Fat Content and Fatty Acid Composition

Total lipid content and FA composition of breast muscle of Kuroilers are presented in Table 6. Total lipid, was not affected (*p* > 0.05) by treatment and sex. Interactions between treatment and sex (*p* < 0.05) were observed for total lipid (CF: 1.93 g; CM: 1.88 g; AF: 2.39 g; AM: 2.15 g; BF: 2.04 g; BM: 1.95 g; ABF: 2.10 g; ABM: 1.85 g).

Research on the effects of prebiotics on fatty acid composition is fairly recent, so generally there is limited information in literature with which to compare the results of this study. Total amount of saturated fatty acids (SFA) and single SFA were not affected (*p* > 0.05) by the treatments. As for single SFA composition (data not shown), the most abundant SFA was palmitic acid (C16:0, ranging from 21.31 to 24.00 %) followed by stearic acid (C18:0, ranging 3.92 to 5.90 %) and several other SFA (C12:0, C14:0, C15:0, C20:0, C22:0) with lower amount (similar or less than 1% each). The total amount of monounsaturated fatty acids (MUFA) was affected (*p* = 0.012) by the treatment; in general, breast muscles of the B group as well as those of the AB group had lower amounts of MUFA compared to C and A groups. This trend was linked to the amount of oleic acid (C18:1 n-9), the most abundant MUFA, which was lower (*p* < 0.01) in B (19.52%) and AB (18.27%) groups compared to C (24.73) and A (24.44%) groups. No significant differences were found for other MUFA (C14:1, C15:1, C16:1, C17:1, C24:1). Meat from the B group displayed a higher (+3.72%) amount of total polyunsaturated fatty acids (PUFA) compared to the control group (*p* < 0.05). The B group also had slightly higher (*p* = 0.096) amount of PUFA compared to the A group, while no significant difference was observed between the B and AB groups (*p* > 0.05). Total amount of n-3 PUFA (C18:3, C20:3, C20:5, C22:5) was higher in B compared to the control (*p* < 0.05) group; while, A and AB had intermediate values (*p* = 0.09). Total n-6 PUFA (C18:2, C18:3, C20:4) was not statistically different among treatment groups. The reduction of total MUFA content and the increase of total PUFA content observed with prebiotics in ovo injected (B group) was also observed by Tavaniello et al. [20] in broiler chickens in ovo injected with different prebiotics. Likewise, Velasco et al. [48] obtained similar results with inulin a fructan prebiotic when administered together with sunflower oil in broiler chickens. Regarding the selected fatty acid ratios, the ratios of PUFA/SFA, n-6/n-3 PUFA, are widely used to evaluate the nutritional value of fat. The n-6/n-3 ratio was lower in B (*p* < 0.01) and AB (*p* < 0.05) groups compared to C and A groups; while PUFA/SFA ratio was similar among experimental groups. Generally, the obtained data showed a particularly lower n-6 to n-3 ratio across the experimental groups due to the higher incidence of n-3 fatty acids. Sirri et al. [46] suggested that the lower n-6 to n-3 ratio is most likely associated with age at slaughter and genotype of the birds used. The PUFA/SFA ratios found in the present study are quite similar to those reported for older birds [49], but lower than those reported by Eleroglu et al. [47] (2.78–3.47). Contrarily, Velasco et al. [48] and Tavaniello et al. [20] found higher PUFA/SFA ratio in the prebiotic groups compared to the control. Considering the current dietary advice, the mean values of the n-6/n-3 ratio found in the present study (ranging from 1.46 to 2.67) are in the ideal range of 1–4; also, the PUFA/SFA values are favorably high (ranging from 0.97 to 1.11). From a nutritional point of view, a higher PUFA/SFA ratio is recommended (i.e., above 0.4).

The results on fatty acid composition of Kuroiler chickens showed a marked difference on the proportion of a few fatty acids in the breast muscle between males and females. In particular, males had higher total MUFA amount compared to females (+2.2%, *p* < 0.05), while the latter displayed higher total PUFA amounts (+1.9%, *p* < 0.05). The n-6/n-3 ratio was slightly higher (*p* = 0.093) in males than in females. The rest of the fatty acids and fatty acid ratios were not affected by sex of the birds. In this study, the birds were slaughtered at 18 weeks of age when the females were starting to lay eggs. These observations are therefore probably due to differences in lipid metabolism mainly associated with hormonal changes that occur with the commencement of egg production in females. In fact, several sex-related differences may be explained by the physiological changes in metabolism in female birds due to egg laying. Scholtz et al. [50] reported that during the laying period, the hepatic synthesis of triglycerides, phospholipids, and cholesterol is generally increased. According to Dal Bosco et al. [51] who obtained similar results in a study of the effect of genotype and slaughter age on fatty acid composition in egg-line and meat-type birds, laying hens seem to have a higher efficiency in long chain fatty acid deposition compared to meat-type chickens. Alessandri et al. [52] noted that the elongation of the fatty acids is partly affected by the oestrogen level which apparently rises when the hens approach or start egg laying, declining slowly by the laying sequence. There were significant treatments x sex interaction effects on some individual fatty acids and total n-3 PUFA (*p* < 0.05; CF: 11.40%; CM: 8.64%; AF: 13.55%; AM: 9.16%; BF: 14.14%; BM: 16.11%; ABF: 11.38%; ABM: 15.68%) as well as n-6/n-3 ratio (*p* < 0.01 CF: 2.20; CM: 3.14; AF: 1.88; AM: 2.83; BF: 1.40; BM: 1.52; ABF: 2.35; ABM: 1.46).

## 4. Conclusions

To the best of our knowledge, this is the first trial of in ovo delivery of prebiotics under field conditions in Uganda, if not the tropics at large. The study has demonstrated that prebiotics with or without antibiotics reduced severity of intestinal lesions and oocyst excretion induced by natural infection with *Eimeria*. Prebiotics protected Kuroiler chickens from coccidia in particular in the first 56 days of age and tended to have a synergistic effect with anticoccidial drug in the management of the disease post-infection in the field. In ovo prebiotic administration without antibiotic (B) increased chicken BWG from the third to sixth week, even though the total BWG was higher in the prebiotic + antibiotic group (AB). In general, slaughter traits and fatty acid profile were positively affected by in ovo prebiotic administration. In particular, groups treated with prebiotics with or without antibiotic displayed a meat with a higher amount of PUFA and total n-3, but lower content of MUFA and n-6/n-3 ratio. It can be assumed that there is a crosstalk between gut microbiome and FA metabolism, however the mechanism linked with the metabolic changes mentioned above are still unclear. Males had significantly higher slaughter weights and better carcass traits compared to females. Nevertheless, further research is needed to increase the knowledge on the effect of in ovo delivery of prebiotic composition on performance and meat quality, with a special emphasis placed on FA profile, of Kuroiler chickens. The results obtained, even if preliminary, confirm the effectiveness of the injection in ovo under sub-optimal environmental conditions. Considering that in Uganda the main challenges to improve poultry production is the prevalence of poultry diseases that threaten the intensification of production, the in ovo technology could be one of a feasible way to improve the health and well-being of chickens and consequently increase the quality and safety of derived food. In the light of the results of the present study, we can say that prebiotic in ovo delivered could reduce or eliminate the use of antibiotics.

## Figures and Tables

**Table 1:** Composition of feed mixtures.

Component (%)	Starter (0–6 Weeks)	Grower (6–14 Weeks)	Finisher (14–18 Weeks)
Maize bran	52.36	64.72	60.6
Sunflower cake	20.94	19.42	18.2
Fish meal	20.94	7.12	18.2
Lime	0	6.27	0
Magic protein ^1^	1.05	1.29	0
NaCl	0.52	0.65	0.6
Mineral-vitamin premix ^2^	0.52	0.52	0.6
Calcium	2.09	0	0.6
Bovanite (Sodium bentonite)	1.57	0	0.6
Meat booster ^3^	0	0	0.6

^1^ Provided per kg of diet: plant protein, 34%; animal protein, 14%; natural minerals, 47.7%; trace elements, 1.8%; vitamins, 2.5%. ^2^ Provided per kg of diet: Vitamin A, 13,000 IU; vitamin D3, 4000 IU; vitamin E, 80 IU; vitamin K, 3 mg; riboflavin, 6.0 mg; pantothenic acid, 6.0 mg; niacin, 20 mg; vitamin B6 2 mg; folic acid, 0.5 mg; biotin, 0.10 mg; thiamine, 2.5 mg; vitamin B12 20 mg; Mn, 120 mg; Zn, 90 mg; Fe, 30 mg; Cu, 10 mg; I, 1.5 mg; Se, 0.2 mg; antioxidants, 100 mg. ^3^ Provided per kg of diet: Metabolizable energy, 11.9%; crude protein, 35.7%; lysine, 1.3%; methionine, 2.03%; tryptophan, 7.3%; crude fiber, 3.7%; threonine, 13.6%; calcium, 02.04; phosphorus, 15.3% and traces of sodium, potassium, xylanase, amylase-protease, antioxidant.

**Table 2:** Effect of the treatment on body weight gain (g) of Kuroiler chickens reared under tropical climatic condition.

Age (Weeks)	Treatment ^1^				SEM	*p*-Values
	C	A	B	AB		
1–3	80.83 ^a^	66.30 ^a,b^	60.83 ^b^	66.60 ^a,b^	2.79	0.035
3–6	157.73 ^B^	192.97 ^b^	245.37 ^A,a^	166.83 ^B^	10.88	0.000
6–9	289.27	308.37	252.87	301.80	8.17	0.040
9–12	350.57 ^A^	296.30 ^a^	226.17 ^B,b^	340.07 ^A^	15.64	0.000
12–15	326.17	404.10	395.73	416.87	22.15	0.531
15–18	498.90	381.80	515.87	474.73	25.29	0.254
1–18	1703.60 ^b^	1649.84 ^c^	1696.84 ^b^	1767.00 ^a^	13.02	0.000

^1^ Treatment: C = Control; A = Antibiotics; B = Bitos; AB = Antibiotics + Bitos. SEM = standard error means. Means in a row with different letters are significantly different at: ^A,B^ *p* < 0.01; ^a,b^ *p* < 0.05.

**Table 3:** Effect of the treatment on oocyt excretion (thousands) per gram of excreta (OPG) in naturally infected Kuroiler chickens.

Age (week)	Treatment ^1^ (OPG in 000)				SEM	*p*-Value
	C	A	B	AB		
6	0.009	0.005	0.000	0.000	0.003	0.281
7	0.027 ^a^	0.017 ^a,b^	0.000 ^b^	0.000 ^b^	0.003	0.019
8	0.070 ^a^	0.016 ^a,b^	0.000 ^b^	0.000 ^b^	0.006	0.015
9	1.143 ^A^	0.508 ^a^	0.039 ^B,b^	0.018 ^B^	0.034	0.000
10	1.373 ^A,a^	0.786 ^A,B,b^	0.466 ^B^	0.473 ^B^	0.049	0.001
11	82.871 ^a^	72.115 ^a,b^	47.701 ^b^	47.534 ^b^	3.252	0.010
12	31.026 ^a^	29.810 ^a^	18.585 ^b^	18.590 ^b^	1.095	0.005
13	15.694 ^A^	10.301 ^B,a^	6.628 ^B,b^	7.518 ^B^	0.339	0.000
14	0.910	0.097	0.016	0.046	0.102	0.040

^1^ C = Control; A = Antibiotics; B = Bitos; AB = Antibiotics + Bitos. SEM = standard error means. Means in a row with different letters are significantly different at: ^A,B^ *p* < 0.01; ^a,b^ *p* < 0.05.

**Table 4:** Effect of the treatment on intestinal lesion score in Kuroiler chickens, reared under tropical climatic conditions, naturally infected with *Eimeria* spp.

Age (Week)	Treatment ^1^				SEM	*p*-Value
	C	A	B	AB		
12	2.67	2.00	1.00	0.67	0.373	0.283
18	1.67 ^A^	0.67 ^A,B^	0.17 ^B^	0.17 ^B^	0.132	0.002

^1^ C = Control; A = Antibiotics; B = Bitos; AB = Antibiotics + Bitos. SEM = standard error means. Means in a row with different letters are significantly different at: ^A,B^ *p* < 0.01.

**Table 5:** Effect of the treatment and sex on **s**laughter traits, pH and WHC of Kuroiler chicken reared under tropical climatic conditions.

Animals, n	Treatment (T) ^1^				Sex (S) ^2^		SEM	*p*-Value		
	C	A	B	AB	F	M				
	6	6	6	6	12	12		T	S	T × S
Body weight	1758.3	1700.00	1783.3	1700.0	1458.3	1987.5	26.2	0.307	0.001	0.724
Carcass weight (g)	1085.3	1013.2	1180.2	1012.8	904.5	1241.2	19.9	0.027	0.001	0.156
Carcass yield (%)	61.87	59.88	65.87	59.58	61.97	62.48	0.98	0.206	0.799	0.504
Breast weight (g)	215.83	237.00	261.00	255.00	214.83	269.58	9.88	0.395	0.014	0.240
Breast yield (%)	19.38 ^b^	23.98 ^a,b^	22.43 ^a,b^	25.40 ^a^	23.96	21.64	0.68	0.036	0.106	0.048
Legs weight (g)	262.83	265.50	314.17	322.67	233.75	348.83	8.90	0.055	0.001	0.385
Legs yield (%)	24.08	26.40	26.83	31.65	26.25	28.23	0.96	0.079	0.319	0.459
Wings weight (g)	91.33 ^b^	107.67 ^a,b^	123.00 ^a^	122.83 ^a^	98.17	124.25	2.99	0.005	0.001	0.028
Wings yield (%)	8.57 ^b^	10.90 ^a,b^	10.52 ^a,b^	12.17 ^a^	11.06	10.01	0.37	0.023	0.172	0.167
Back + neck weight (g)	229.00	197.67	261.50	248.33	194.25	274.00	7.78	0.053	0.001	0.404
Back + neck yield (%)	21.25	19.47	22.08	24.80	21.80	22.00	0.74	0.123	0.894	0.744
pH	5.70	5.71	5.67	5.65	5.67	5.68	0.01	0.246	0.490	0.185
WHC (%)	10.24	9.85	11.05	9.47	10.15	10.15	0.45	0.637	0.999	0.831

^1^ C = Control; A = Antibiotics; B = Bitos; AB = Antibiotics + Bitos. ^2^ Sex: F = Female; M = Male. SEM = standard error means. Means in a row with different letters are significantly different at: ^a,b^ *p* < 0.05.

**Table 6:** Effect of the treatment and sex on total lipid (g/100 g) and fatty acid profiles (% of total fatty acids) of breast muscle from Kuroiler chickens reared under tropical climatic conditions.

Animals, n	Treatment (T) ^1^				Sex (S) ^2^		SEM	*p*-Value		
	C	A	B	AB	F	M		T	S	T × S
	6	6	6	6	12	12				
Total lipid	1.90	2.27	2.00	1.98	2.11	1.96	0.07	0.346	0.146	0.044
∑ SFA	36.65	35.59	35.80	38.47	36.78	36.47	0.522	0.231	0.767	0.787
∑ MUFA	27.57	27.51	24.71	24.15	24.89	27.07	0.405	0.012	0.016	0.131
∑ PUFA	35.78 ^b^	36.91 ^a,b^	39.50 ^a^	37.38 ^a,b^	38.32	36.46	0.334	0.009	0.013	0.309
PUFA/SFA	0.98	1.05	1.11	0.97	1.05	1.00	0.024	0.182	0.360	0.874
∑ n-3	10.02 ^b^	11.35 ^a,b^	15.12 ^a^	13.51 ^a,b^	12.62	12.39	0.489	0.010	0.819	0.022
∑ n-6	25.76	25.56	21.97	23.87	24.01	24.57	0.625	0.156	0.661	0.100
n6/n3	2.67 ^A,a^	2.35 ^A,a^	1.46 ^B^	1.90 ^b^	1.96	2.24	0.078	0.000	0.093	0.002

^1^ C = Control; A = Antibiotics; B = Bitos; AB = Antibiotics + Bitos; ^2^ F = Female; M = Male. SFA = saturated fatty acids; MUFA = monounsaturated fatty acids; PUFA = polyunsaturated fatty acids. SEM = standard error means. Means in a row with different letters are significantly different at: ^A,B^ *p* < 0.01; ^a,b^ *p* < 0.05.
